# Computational fluid dynamic modeling of the lymphatic system: a review of existing models and future directions

**DOI:** 10.1007/s10237-023-01780-9

**Published:** 2023-10-30

**Authors:** Tharanga D. Jayathungage Don, Soroush Safaei, Gonzalo D. Maso Talou, Peter S. Russell, Anthony R. J. Phillips, Hayley M. Reynolds

**Affiliations:** 1 Auckland Bioengineering Institute, The University of Auckland, Auckland, New Zealand; 2 School of Biological Sciences, The University of Auckland, Auckland, New Zealand; 3 Surgical and Translational Research Centre, Department of Surgery, Faculty of Medical and Health Sciences, The University of Auckland, Auckland, New Zealand

**Keywords:** Lymphatic system, Mathematical modeling, Computational fluid dynamics, Lymphatic vessels

## Abstract

Historically, research into the lymphatic system has been overlooked due to both a lack of knowledge and limited recognition of its importance. In the last decade however, lymphatic research has gained substantial momentum and has included the development of a variety of computational models to aid understanding of this complex system. This article reviews existing computational fluid dynamic models of the lymphatics covering each structural component including the initial lymphatics, pre-collecting and collecting vessels, and lymph nodes. This is followed by a summary of limitations and gaps in existing computational models and reasons that development in this field has been hindered to date. Over the next decade, efforts to further characterize lymphatic anatomy and physiology are anticipated to provide key data to further inform and validate lymphatic fluid dynamic models. Development of more comprehensive multiscale- and multi-physics computational models has the potential to significantly enhance the understanding of lymphatic function in both health and disease.

## Introduction

The lymphatic system is a complex and critical component of the circulatory system (Selahi and Jain 2022). Its primary function is to maintain fluid and immune homeostasis, while also playing a key role in the absorption of lipids from the intestine (Bernier-Latmani and Petrova 2017). The lymphatic system achieves this by transporting interstitial fluid, macromolecules, and cells back to the circulation through lymphatic vessels, which are located throughout most parts of the body.

Computational modeling is a powerful tool that can be utilized to further understand the lymphatic system through simulation at multiple spatial scales, including at the molecular, cellular, tissue, and organ level (de Bono et al. 2013; de Bono and Hunter 2012; Safaei et al. 2016, 2018; Shahidi et al. 2021, 2022). However, computational models of the lymphatics are much less common than cardiovascular models (Safaei et al. 2016, 2018; Niederer et al. 2019). Progress has been impeded by historical gaps in anatomical and functional knowledge, which is partially due to the challenge of visualizing a unidirectional system that contains clear fluid. This means the entire lymphatic system cannot be visualized by a single injection of a contrast agent (Munn and Padera 2014).

This review summarizes computational models of the lymphatic system with a focus on computational fluid dynamic (CFD) models. It summarizes recent models that have leveraged advances in computational power and numerical methods. It is, however, acknowledged that there have been various earlier efforts in the field of lymphatic mathematical modeling dating back several decades (Horikoshi et al. 1987; Miller and Seale 1985; Taylor 1981). This review advances on prior reviews (Margaris and Black 2012; Moore and Bertram 2018) by including models from the last five years and by summarizing the existing limitations and gaps that have hindered further development in this field to date. Addressing these gaps will assist progress in the development of comprehensive multiscale computational models of the lymphatic system to match those already developed for the cardiovascular system. It will also provide additional in silico tools to enhance understanding of normal lymphatic function, lymphatic-targeted drug delivery, and a range of conditions such as lymphedema, cancer metastasis, and critical illness (Windsor et al. 2022).

## Background

**Fig. 1:**
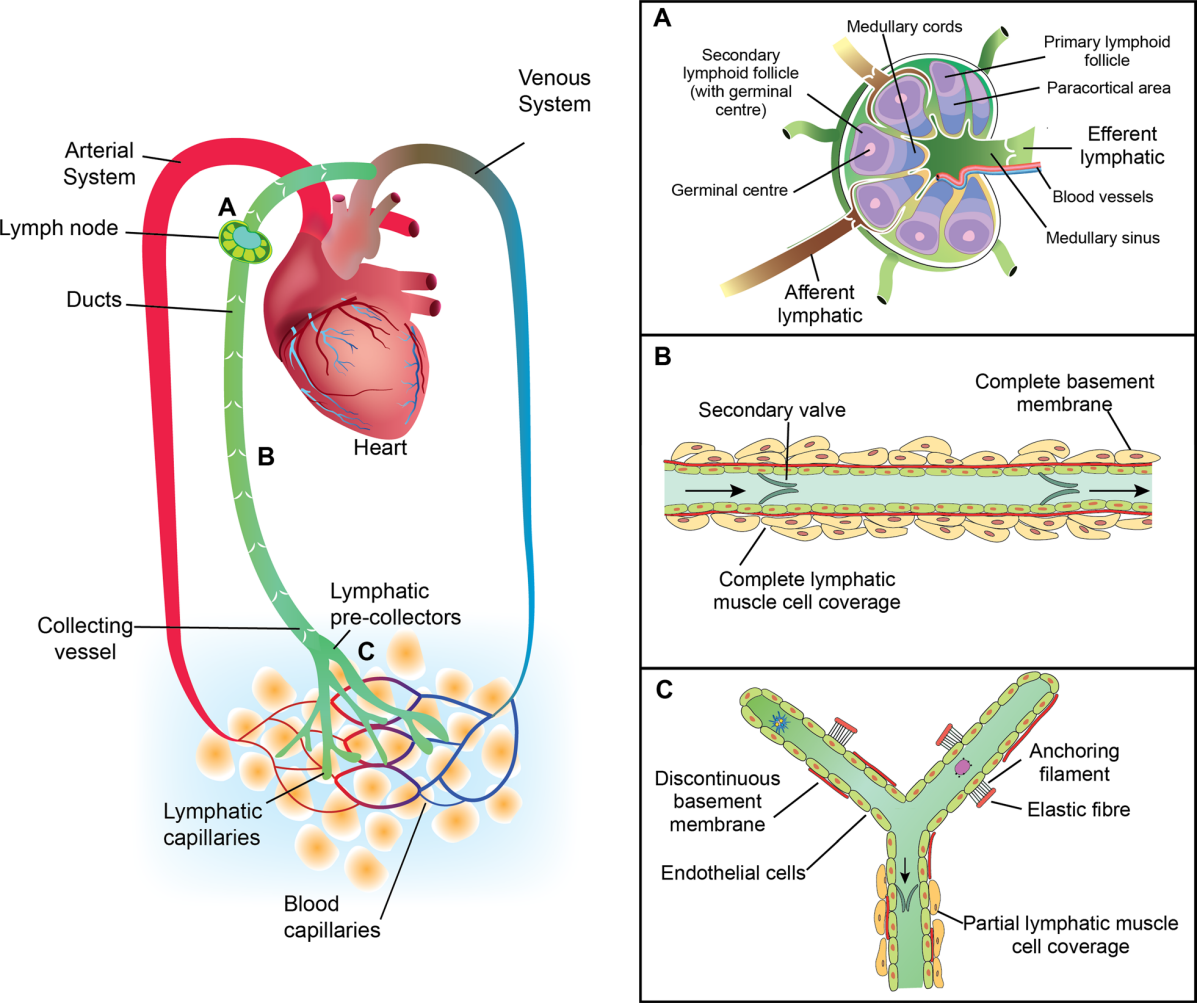
Graphical representation of the lymphatic and cardiovascular systems, showing a representative **a** lymph node, **b** lymphatic collecting vessel, **c** initial lymphatics and pre-collecting vessels

Lymphatic vessels are arranged in a hierarchy, beginning as blind-ended capillaries, which form a mesh-like network called the initial lymphatics that then progress into pre-collectors, collecting vessels, lymphatic trunks, and finally ducts (Fig. 1). Collecting vessels are further divided into afferent (pre-nodal) and efferent (post-nodal) vessels depending on their location relative to lymph nodes (Fig. 1a) (Cooper et al. 2016).

Lymph fluid is returned to the cardiovascular system via the thoracic duct or right lymphatic duct into the central veins of the neck, or via other lympho-venous communications, which are most common within lymph nodes (O’Hagan et al. 2021). Lymph transport generally occurs against a pressure gradient and therefore requires either extrinsic forces such as muscle movement and arterial pulsations, or intrinsic pumping by lymphatic muscle cells within the vessel wall (Scallan et al. 2016).

The anatomical structure of each component of the lymphatic system and its surroundings contributes to its function. The initial lymphatics are connected to the extracellular matrix (ECM) of the interstitium (consisting of ECM and interstitial fluid) via anchoring filaments (Fig. 1c) (Leiderman et al. 2006). Blood capillary filtration occurs along the entire length of the microvascular bed in most tissues under steady-state conditions in accordance with the revised Starling principle (Levick and Michel 2010). High rates of filtration can increase the total interstitial volume by expanding the ECM because of its poroelastic properties (Leiderman et al. 2006). Current understanding is that initial lymphatics maintain tissue fluid homeostasis by absorbing all fluid that is filtered from blood capillaries (Levick and Michel 2010).

Lymphatic vessel walls are comprised of endothelial cells, lymphatic muscle cells (for pre-collectors (partial coverage) and collecting vessels), and connective tissue (Fig. 1b, c). Valves are also present to facilitate fluid flow. ‘Primary valves’, formed by the overlapping endothelial cells of initial lymphatics, allow fluid entry into the initial lymphatic vessel lumen but prevent fluid escape. ‘Secondary valves’ are situated within the vessel lumen of pre-collectors and collecting lymphatic vessels and prevent retrograde lymph flow. They are usually bicuspid, but tricuspid and monocuspid valves are also possible (Breslin et al. 2019).

Lymphatic collecting vessels act as both pumps and conduits, with the lymphangion being the basic functional unit located between two secondary valves (Fig. 1b). Lymphatic muscle cells of collecting vessels display both sustained tonic contractions and rhythmic phasic contractions. Tonic contractions provide baseline tension and prevent backflow by ensuring tight apposition of valve leaflets, and phasic contractions actively pump the lymph to the next lymphangion in series. The characteristics of both tonic and phasic contractions depend on a complex interplay between transmural pressure (preload and afterload), shear stress, and endogenous or exogenous molecular agents (Scallan et al. 2016).

The organization of lymphatic networks within various organs, such as intestine, kidney, lung and heart, depends on the functional demands of the tissue, leading to both shared features and unique configurations in lymphatic network structure (Breslin et al. 2019). In the brain, meningeal lymphatics provide a vital pathway for the removal of waste products and toxins, draining cerebrospinal fluid and immune cells from the central nervous system to the peripheral lymphatic system (Louveau et al. 2015; Semyachkina-Glushkovskaya et al. 2023).

## Methods

A literature search of original research articles studying local (tissue scale) and global (lymphatic system scale) lymphodynamics up to August 2023 was conducted using the PubMed and Web of Science online databases and the keywords: ‘Lymphatic system’, ‘Lymph’,‘Computational’, ‘Numerical’, ‘Computer Assisted’, ‘Model’, ‘Biological transport’, 'Biofluid’, ‘Fluid’, ‘Flow’, ‘Interstitial flow’, ‘Interstitial pressure’, ‘Interstitial fluid', ‘Lymph node’ and ‘Lymphedema’.

## Existing computational models of the lymphatics

The above literature search yielded 256 publications from PubMed and 249 publications from Web of Science. A total of 56 publications were then manually identified as relevant studies focusing on lymphodynamics. Table 1 summarizes the 56 studies, with 43 (77%) of them published in the last decade.

**Table 1:** Summary of existing computational models of the lymphatic system

Authors	Year	Model type	Species	Anatomical site	I	IL (PV)	PC	CV (SV)	LN	Validation
Drake et al. (1991)	1991	0D	Sheep	Lung				✓		
Venugopal et al. (2007)	2007	0D	Cow	Mesentery				✓ +		Ex Vivo
Quick et al. (2007)	2007	0D	Cow	Mesentery				✓ +		Ex Vivo
Quick et al. (2008)	2008	0D	Cow	Mesentery				✓ +		Ex Vivo (Venugopal et al. 2007)
Venugopal et al. (2009)	2009	0D	Cow	Mesentery				✓		Ex Vivo (Venugopal et al. 2007)
Bertram et al. (2011)	2011	0D	Rat	Mesentery				✓		
Bertram et al. (2014)	2014	0D	Rat	Mesentery				✓ +		Ex Vivo (Davis et al. 2011)
Jamalian et al. (2016)	2016	0D	Rat	Mesentery				✓ +		
Bertram et al. (2016a, b)	2016	0D	Rat	Mesentery				✓ +		
Caulk et al. (2016)	2016	0D	Rat	Thoracic duct				✓ +		
Jamalian et al. (2017)	2017	0D	Rat	Mesentery				✓ +		Ex Vivo
Bertram et al. (2017)	2017	0D	Rat	Mesentery				✓ +		Ex Vivo
Bertram et al. (2018)	2018	0D	Rat	Mesentery				✓ +		Ex Vivo
Bertram et al. (2019)	2019	0D	Rat	Mesentery				✓ +		
Ngo et al. (2019)	2019	0D	Human	Lung	✓	✓ +		✓		
Ikhimwin et al. (2020)	2020	0D	Rat	Mesentery	✓	✓ +	✓	+		
Morris et al. (2021)	2021	0D	Rat	Mesentery				✓ +		Ex vivo (Zawieja 2009)
Ashworth et al. (2023)	2023	0D	Human	Lung	✓	✓ +				
Reddy et al. (1977)	1977	1D	Human	Whole body				✓		
Reddy and Patel (1995)	1995	1D	–	–		✓ +				
MacDonald et al. (2008)	2008	1D	Cow	Mesentery				✓ +		Ex Vivo
Contarino and Toro (2018)	2018	1D	Rat	Mesentery				✓ +		
Savinkov et al. (2020)	2020	1D	Human	Whole body				✓		
Lavrova and Postnikov (2021)	2021	1D	Fish	Brain	✓	✓				
Mendoza and Schmid-Schöenbein (2003)	2003	2D	Rat	–		✓ +				
Galie and Spilker (2009)	2009	2D	Rat	–	✓	✓ +				
Roose and Swartz (2012)	2012	2D	Rat	Tail	✓	✓ +				
Heppell et al. (2013)	2013	2D	Human	–	✓	✓ +				In vivo (Ikomi et al. 1996)
Heppell et al. (2015)	2015	2D	Human	–	✓	✓ +				
Razavi et al. (2017)	2017	2D	Rat	Tail				✓		In vivo
Li et al. (2019)	2019	2D	﻿–	﻿–	✓	✓		✓ +		
Ikomi and Hiruma (2020)	2020	2D	Rat	–	✓					Ex vivo
Elich et al. (2021)	2021	2D	Rat	Mesentery				✓ +		Ex vivo (Davis et al. (2011), Davis et al. (2012))
Li et al. (2022)	2022	2D	﻿–	–	✓	✓		✓ +		
In et al. (2021)	2021	2D	﻿–	Micro-Fluidic				✓ +		In vitro
Giantesio et al. (2022)	2022	2D	﻿–	﻿–					✓	
Grotberg and Romanò (2023)	2023	2D	Human	Lung	✓	✓ +				
Rahbar and Moore Jr (2011)	2011	3D	Rat	Mesentery				✓		
Wilson et al. (2013)	2013	3D	Rat	Mesentery				✓ +		In vivo (Bohlen et al. 2011)
Jafarnejad et al. (2015)	2015	3D	Mouse	Popliteal					✓	
Wilson et al. (2015)	2015	3D	Rat	Mesentery				✓ +		
Cooper et al. (2016)	2016	3D	Dog	Popliteal					✓	In vivo (Adair and Guyton 1985)
Ballard et al. (2018)	2018	3D	Rat	Multiple				+		Ex vivo
Wilson et al. (2018)	2018	3D	Rat	Mesentery				✓ +		
Possenti et al. (2019)	2019	3D	Human	Mesentery	✓	✓				
Bertram (2020)	2020	3D	Rat	Mesentery				+		
Jayathungage Don et al. (2021)	2021	3D	Rat	Tail	✓	✓ +	✓	✓		In vivo (Swartz et al. 1999)
Han et al. (2021)	2021	3D	Human	Skin	✓	✓ +				
Wolf et al. (2021)	2021	3D	Rat	Mesentery				✓ +		Ex Vivo (Davis et al. 2012)
Tretiakova et al. (2021)	2021	3D	Dog	Popliteal					✓	In vivo (Adair and Guyton 1985)
Sethukha and Tretiakova (2022)	2022	3D	–	–					✓	
Han et al. (2023a)	2023	3D	Human	Skin	✓	✓ +				
Han et al. (2023b)	2023	3D	Human	Skin	✓	✓ +				
Wolf et al. (2023)	2023	3D	Rat	Mesentery				✓ +		
Bertram and Davis (2023)	2023	3D	Rat	Multiple				✓ +		Ex vivo
Adeli Koudehi et al. (2023)	2023	3D	Mice	Skin	✓			✓ +		

I, interstitium, IL, initial lymphatics, PV, primary valves ( +), PC, pre-collectors, CV, collecting vessels, SV, secondary valves ( +), LN, lymph node

Studies were divided into two main categories: lumped parameter (zero-dimensional (0D)) models and continuum models (separated further into 1D, 2D, and 3D models). Table 1 provides the modeled species, anatomical site, and structural components of the lymphatic system considered in each model. The structural components were divided into interstitium (I), initial lymphatics (IL), primary valves (PV), pre-collectors (PC), collecting vessels (CV), secondary valves (SV), and lymph nodes (LN). Validated models were identified, and the source and type of experimental data used in the validation process specified, although, in some cases where publications were based on experimental work, the source was not explicitly stated.

**Fig. 2:**
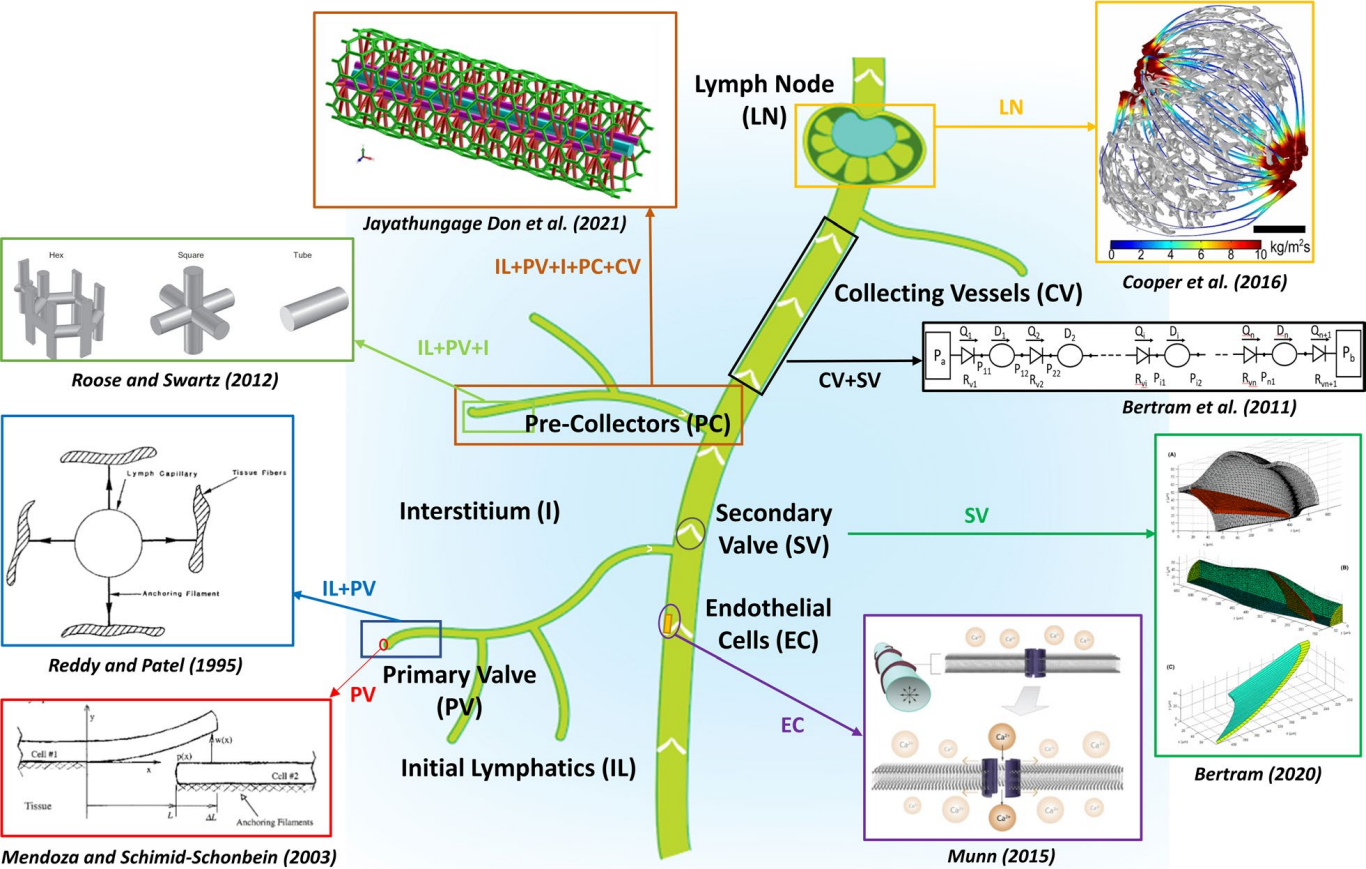
Key studies that have focused on modeling various structural components of the lymphatic system. IL, initial lymphatics; PV, primary valves; PC, pre-collectors; CV, collecting vessels; SV, secondary valves; E, endothelial cell; I, interstitium; LN, lymph nodes

Figure 2 schematically shows each of the main structural components of the lymphatic system and highlights some key studies for each component. Some models spanned multiple components of the lymphatic system, while many focused on a single component, such as the collecting vessels. For example, Bertram et al. (2011) focused on the larger collecting vessels to understand the pumping mechanism of lymphangions, while Ikhimwin et al. (2020) and Jayathungage Don et al. (2021) considered multiple parts of the lymphatic system to understand the drainage through the lymphatic vessel network hierarchy. Some studies reviewed key aspects of lymphatic physiology to consider in computational modeling approaches, such as Munn (2015), who summarised mechanobiological control mechanisms in the lymphatic vessels.

### Initial lymphatics and pre-collectors

Lymphatic drainage is susceptible to pressure and volume changes in the interstitium, and many studies have included the interstitium in their models (Table 1). Some studies modeled fluid drainage through the interstitium using Darcy’s law (Ashworth et al. 2023; Gortberg and Romano (2023); Han et al. 2023a, b; Heppell et al. 2013, 2015; Ikhimwin et al. 2020; Possenti et al. 2019), which neglects viscous and inertial effects. However, viscous effects can be present in the interstitium under normal conditions (Moore and Bertram 2018). Other studies have considered viscous terms when modeling the interstitial fluid flow (Jayathungage Don et al. 2021; Roose and Swartz 2012), which has resulted in higher flow velocities in the interstitium immediately adjacent to the initial lymphatic vessel walls and lower velocities further away.

#### Lumped models

A lumped parameter model is a simplified mathematical model used to describe the behavior of a complex system by representing it as a set of discrete components, or “lumped parameters”, that interact with each other. Three out of the eighteen studies that modeled the initial lymphatics developed lumped parameter models (Ashworth et al. 2023; Ikhimwin et al. 2020; Ngo et al. 2019) (Table 1).

A comprehensive approach, taken by Ikhimwin et al. (2020), was a significant development. They modeled lymphodynamics through a hierarchy of vessels, including the initial lymphatics, pre-collectors, and collecting vessels, surrounded by the interstitial space. This was the first published model to consider initial and pre-collecting lymphatic vessel components, including primary and secondary valves and the interstitium. Importantly, Ikhimwin et al. (2020) used confocal microscopy imaging data to define the initial lymphatic branching structure, inter-valve length in the pre-collectors, vessel diameter, and vessel length. They adapted an equation developed for lymphangions (Bertram et al. 2011; Jamalian et al. 2016) in which the flow was modeled using the Poiseuille formulation. Importantly, they used a nonlinear primary valve resistance function, based on the primary valve model of Galie and Spilker (2009) and a sigmoidal function to represent the secondary valve response originally developed by Bertram et al. (2014). As there were no data available to quantify the primary valve density, both uniform and downstream-increasing density values were investigated.

Ikhimwin et al. (2020) showed that increased initial lymphatic length increases lymph formation, which differed from Reddy and Patel (1995). The main contribution of Ikhimwin’s study was to provide a platform for understanding the interaction between multiple phenomena, such as vessel stiffness, primary valve resistance, interstitial resistance, intravascular resistance, and inflammation, in relation to lymphatic drainage. They assumed a linear pressure and diameter relationship and nonlinear valve resistance for primary valves in the absence of experimental data.

Ashworth et al. (2023) published a pulmonary lymphatic model to understand the interaction between cardiac dysfunction and pulmonary lymphatic function. Their focus was on the pulmonary capillary blood pressure resulting from chronic high left atrial pressure (LAP) caused by left heart disease, which is known to lead to pulmonary edema. Ashworth et al. (2023) developed a new model of lymphatic capillary absorption and combined it with existing models of lung ventilation, perfusion, and mechanics. Lymphatic capillaries, interstitial spaces, and initial lymphatic vessels were each considered, and lymph fluid flow was modeled by opening pores between the interstitium and initial lymphatics. Their results predicted that pulmonary edema could form when LAP increased to 25 mmHg.

#### Continuum models

The first continuum model (1D) of initial lymphatics was developed by Reddy and Patel (1995) (Fig. 2). They assumed that lymph flow was axisymmetric and dependent on the pressure difference between the interstitium and the interior of the initial lymphatic vessel. They also assumed that lymphatic vessels were hyperplastic submerged vessels with an external attachment to the interstitium via anchoring filaments, and they incorporated a model of the primary valves. Their results concluded that collecting vessel contractions affect the resultant flow rate within the initial lymphatic vessels, supporting the later hypothesis that lymph flow forms via suction of collecting lymphatic vessels (Jamalian et al. 2017). Reddy and Patel’s model also showed that increasing the length of the initial lymphatics after a certain length does not improve the absorption. In their mathematical modeling, the 1D Poiseuille flow equation was solved by considering force balance at the vessel wall, accounting for the vessel’s hoop stress and the external pressure and force exerted by the anchoring filaments. Due to a lack of quantitative anatomical data, their model required many assumptions to define parameter values for the initial lymphatics. For example, the elasticity of initial lymphatic vessels was estimated to be ten times the elasticity of blood capillaries, which had a known value in the literature.

Recently, there has been a surge in meningeal lymphatic research, with several studies targeting the role of meningeal lymphatic vessels in cerebrospinal fluid clearance, employing diverse methodologies (Da Mesquita et al. 2018; Lavrova and Postnikov 2021). Lavrova and Postnikov (2021) focused on investigating the dynamic processes within meningeal lymphatic capillaries, utilizing experimental data, including measurements of meningeal lymphatic vessel contours, and tracking neutrophil transport in a zebrafish model. Their work demonstrated the ability of a 1D numerical model to replicate biophysical experimental data, specifically the transport of neutrophils by lymph flow.

Heppel et al. (2013) developed an initial lymphatic model considering primary valves, blood capillary filtration, and the interstitial space. An important contribution of both models was the inclusion of capillary blood filtration and the resulting interstitial flow. Heppel et al. (2013) found that the fluid flux per unit area through the primary valve was similar to the primary valve model of Mendoza and Schimid-Schoenbein (2003). They also validated the volumetric flow rate through the initial lymphatic lumen against published experimental data obtained from a rabbit hind leg (Ikomi et al. 1996).

In a subsequent publication, Heppel et al. (2015) investigated fluid flow through a deformable interstitium and modeled uptake into the lymphatic system. Here, they used the hypothesis that primary valves act via a ‘sliding door’ mechanism. This mechanism acts by overlapping endothelial cells along the initial lymphatic vessel walls opening with the expanding interstitial space, which allows fluid to enter the lymphatic lumen paracellularly. Model simulations by Heppel et al. (2015) reached a steady state when there was a constant pressure difference between the blood and lymphatic capillaries. In addition, they found that pressure fluctuations within the lymphatic system were significantly smaller than the pressure difference between blood and lymphatic vessels and that the steady-state solution accurately reflected the tissue’s state under physiological conditions.

Roose and Swartz (2012) used homogenization theory to find the optimal initial lymphatic capillary network structure for facilitating interstitial fluid uptake (Fig. 2). Homogenization is a method used to study a system at two spatial scales, such as the tissue scale (macro) and the lymphatic capillary scale (micro). Roose and Swartz (2012) modeled lymphatic capillary flow using Navier–Stokes equations and interstitial flow using Darcy’s law. They then used the homogenization method to derive a leading-order model, which is valid on the macroscale, and retained the important microstructural effects, such as capillary permeability. They based their study on rodent tails and showed that hexagonal lymphatic vessels, a pattern observed in both rat and human skin, are optimal for draining interstitial fluid (Schmid-Schonbein 1990).

Possenti et al. (2019) developed a coupled hybrid model, integrating a localized sink term for the lymphatic system, a 1D blood capillary network, and the surrounding 3D interstitium. The sink term was used to represent localized reductions in fluid quantity due to absorption by lymphatic capillaries. To drive the flow, they employed a nonlinear interstitial pressure function and assessed both normal and pathological drainage conditions. Their findings demonstrated the impact of reduced localized lymphatic drainage on spatial interstitial pressure changes within the 3D model. This novel approach shed light on the intricate interplay between fluid dynamics and lymphatic function, contributing valuable insights to the field of lymphatic research.

Next, Ikomi and Hiruma (2020) investigated the physiological significance of irregular cross-sectional shapes in the initial lymphatics. Their comprehensive study included histological experiments and fluorescence imaging in rats to assess stretch-induced morphological changes. They also conducted mechanical investigations of initial lymphatic vessel gap expansion under tension and used finite element modeling (FEM) to simulate changes in the cross-sectional areas of the initial lymphatics. When the tissue was stretched, they observed that irregularly shaped initial lymphatics had a higher luminal volume than round-shaped ones. Fluorescent images and histological studies confirmed the expansion of initial lymphatics due to stretching. Importantly, they also experimentally investigated massage-induced lymph formation and cross-sectional changes in the initial lymphatics. They used their experimental data to validate their FEM model and established the relationship between lymph formation rate and massage frequency of the surrounding tissue.

The most recent 2D model was presented by Grotberg and Romanò (2023) who developed a microvascular model simulating fluid transport in the alveolar septa, with a specific focus on pulmonary edema. Their model suggested that interstitial pressures in the alveolar septa were significantly higher than physiological values reported in the literature. These higher-pressure gradients in the septal interstitial space play an essential role in driving the flow of fluid away from the alveolar sacs and toward the distant lymphatic vessels. Furthermore, Grotberg and Romanò (2023) used the 2D model to compare both normal and pathological conditions associated with fluid transport in this context.

Jayathungage Don et al. (2021) developed a 3D lymphatic drainage model based on rat tail lymphatic anatomy with surrounding interstitium to model the initial lymphatics, pre-collectors, and collecting vessels (Fig. 2). The Navier–Stokes equation and Darcy’s law were used to model lymphatic and interstitial flows. Lymphatic primary valves were modeled using wall permeability, and the scalar transport equation was utilized to simulate drug absorption and transport. By solving equations in a 3D geometrical model, drug transport efficacy could be estimated by varying drug bolus shape, drug particle size, and flow regimes.

Han and co-workers conducted similar 3D studies to estimate the lymphatic uptake of injected biotherapeutics into human skin. First, Han et al. (2021) modeled the lymphatic absorption and blood perfusion as source terms in the continuity equations. Later, Han et al. (2023a) improved their model by explicitly adding blood and lymphatic 3D discrete continuum networks to the model. An arbitrary tree generation algorithm was used to create blood and lymphatic capillaries, giving intertwined vessels, in contrast to recent anatomical findings that suggest skin lymphatic capillaries are not arranged in this manner (Wang et al. 2014).

Han et al. (2023a) used Darcy’s law to model flow in the interstitium and Starling’s law to model flow in blood and lymphatic vessels. The transport (convection and diffusion) equation was then used to solve the drug transport. Han et al. (2023a) showed that lymphatic uptake following subcutaneous administration significantly affects macroscopic drug absorption. They also found that the injection depth does not affect lymphatic uptake, which contrasts with the findings of Jayathungage Don et al. (2021). Following this, Han et al. (2023b) later improved their transport model to investigate the effects of the drug radius. Additionally, they enhanced the model’s time-efficiency, reducing the computational expense that was a limitation in the prior model, so it could be used in a clinical setting.

#### Primary valve models

Ten studies have modeled the primary valves within the initial lymphatics. Mendoza and Schmid-Schönbein (2003) developed a 2D model of the primary valves where the opening and closing mechanisms were based on bending of two adjacent endothelial cells (Fig. 2). One endothelial cell extension was assumed to be fixed and the adjacent endothelial cell was free. Due to the acting pressure forces, the free end could bend inward to create a gap. Modeling was carried out using a solid mechanics approach, which considered elastic deformation of the free endothelial cell. Galie and Spilker (2009) refined this model and created a finite element model (FEM) that considered complex mechanics such as fluid flow within a porous interstitium and primary valve function, which was modeled by bending a thin cell. Following these models, two hypotheses were proposed to explain the mechanism of fluid uptake into initial lymphatics by Heppel et al. (2013, 2015). Based on the Mendoza and Schmid-Schönbein model, the first hypothesis proposes that lymphatic valves bend, due to the hydrodynamic pressure gradient between the interstitium and the lymphatic lumen. The second hypothesis is the ‘sliding door’ mechanism mentioned above (Sect. 4.1.2). Most published studies use the bending valve hypothesis, which was the only one available until the introduction of the sliding door hypothesis (Galie and Spilker 2009; Mendoza and Schmid-Schoenbein 2003; Reddy and Patel 1995).

### Collecting vessels

Most computational models of the lymphatic system have focused on collecting vessels, with the majority being lumped parameter models (Table 1). Vessel tone, phasic contractions, and secondary valve dynamics are the main functional aspects to capture. The following summarizes existing models of collecting vessels developed using lumped parameter and continuum models.

#### Lumped parameter models

Drake et al. (1991) were the first to model collecting vessels using a simple electrical analog based on a linear pressure–flow relationship. They aimed to isolate the influence of active pumping on the resultant lymph flow, which is a combination of active and passive forces. They used published experimental data on sheep lung lymphatic outflow pressure versus flow, which included active and passive mechanisms, as well as data on volume versus transmural pressure of isolated contracting bovine lymphatics. They also compared data between anesthetized sheep (with suppressed lymphatic contractions) and awake sheep (with no suppression of lymphatic contractions). Their linear pressure-resistance circuit model agreed well with the experimental data, and the differences between anesthetized and awake sheep were used to estimate the contribution of active pumping to overall lymph flow.

After a gap of many years, the next lumped parameter model was published by Quick et al. (2007). They used a simplified system of algebraic equations (based on linearized Navier–Stokes equations) to develop a relatively complex model based on bovine lymphatic vessels. Computational methods were adapted from the time-varying elastance concepts utilized to study cardiovascular dynamics to understand lymphangion contractility and to predict flow rates at positive and negative axial pressure gradients. Their model showed that contraction increases flow with normal axial pressure gradients (i.e., higher pressure peripherally). When the pressure gradient was reversed, lymph fluid traveled passively, and therefore, active pumping was inhibited and could only occur when the lymphangion inlet pressure was less than the outlet pressure. At this point, the valves are fully open. This model simulated the dual function of lymphatic collecting vessels, i.e., a pump and a conduit. However, it incorporated many assumptions and neglected temporal contractile variability.

Venugopal et al. (2007) investigated the effects of multiple coordinated lymphangions connected in series on the mean lymph flow using the same mathematical models developed by Quick et al. (2007). Coordination between adjacent lymphangions is thought to be an important phenomenon to simulate for overall lymph flow. However, Venugopal et al. (2007) considered different time delays between lymphangions and concluded that coordination of lymphangion contraction has little impact on mean output flow, meaning that individual lymphangions have the flexibility to adapt to local conditions. They also conducted ex vivo experiments in rats and guinea pigs and observed lymphatic muscle cell discontinuities across valves that separate adjacent lymphangions, which has functional importance for lymphatic pumping. However, their model lacked valves between lymphangions, affecting its validity.

Quick et al. (2008) published another model, which used a first-order approximation for the pressure–flow relationship to consider spontaneously contracting lymphangions. Here, they applied Poiseuille’s law rather than the linearized Navier–Stokes equation used in their previous publication. They derived an algebraic formula for predicting lymphangion flow based on physical laws and lymphangion properties. This was an important contribution as it allows one to empirically relate structural and functional properties. Their developed mathematical model has been used in subsequent studies (Bertram et al. 2011, 2014; Ikhimwin et al. 2020), which added physiological data based on secondary valve function and inhibition of flow in lymphangions.

Venugopal et al. (2009) published a model using Poiseuille’s law to relate lymphangion structure to its function. Their model (based on animal data) illustrated that lymph flow is optimized at a certain lymphangion length and a symmetrical network of lymphatic vessels. The optimal lymphangion lengths for 0.1 and 2.0 cm H_2_0 transmural pressure were 0.1 and 2.1 cm, respectively. However, they only considered length as a structural parameter.

From 2011 onwards, Bertram and colleagues contributed significantly to the field of lymphatic modeling using lumped parameter computational models (Bertram et al. 2011, 2014, 2016a, b, 2017, 2018; Bertram 2020; Ikhimwin et al. 2020; Bertram and Davis 2023) (Fig. 2). These models were informed by ex vivo collecting lymphatic vessel experiments with the aim of explaining and validating the available data. Pioneering experimental work by Davis et al. (2011) that considered valve gating of an isolated collecting vessel was particularly important for informing these models. Bertram et al. (2011) developed a nonlinear complex ordinary differential equation (ODE) model with a chain of multiple lymphangions, which differed from all previous models by incorporating lymphangion pumping. The previous models (Quick et al. 2007; Venugopal et al. 2007, 2009) utilized a different approach based on specifying changes in lymphangion stroke volume. The lymphangion contraction frequency was based on experimental data. Bertram et al. (2011) considered additional lumped components in the model, such as peristaltic motion (pumping action), to model physiological responses. Like previous approaches (Quick et al. 2007; Venugopal et al. 2007, 2009), they used the Poiseuille flow equation due to the minute flow velocities. However, the implementation of the force balance equation of the wall considering lymphatic muscle cell forces, passive properties of wall stiffening, and vessel compliance was a significant improvement.

Another original contribution from Bertram et al. (2011) was the inclusion of pressure-dependent secondary valve resistance. In their model, they considered active contraction and passive elasticity terms, resulting in wall stiffening at high internal pressures and loss of compliance at higher external pressures, which agrees with physiological observations. They also demonstrated that coordinated pumping between lymphangions is inefficient. As mentioned above, Venugopal et al. (2009) supported their findings with in vitro experimental data, but the bovine lymphatic vessels that they used did not have valves.

Following this, Bertram and co-workers published a series of studies (Bertram et al. 2016a, b; 2018) that improved their initial models of secondary valves and lymphangions. These subsequent models used a highly nonlinear passive pressure–diameter relationship (Moore Jr. and Bertram 2018). Importantly, because phasic contractions of the vessel wall affect the function of the lymphangion, these periodic contractions were implemented by imposing an activation waveform that depended on the tension of the instantaneous muscle length. This prescribed activation waveform represents the explicit time-dependent active circumferential tension.

Bertram et al. (2014) later improved their valve model by considering experimentally measured valve properties, including hysteresis, which accounts for the deviation of valve opening and closing pressure drop thresholds, leading to a biased tendency to remain open. Their focus was to develop a numerical scheme that could handle this improved valve function. Previously, Bertram et al. (2011) had not considered the refractory period of the lymphangion (the period following a contraction in which the muscle is incapable of a subsequent contraction) or the delay between contractions of adjacent lymphangions. However, when Bertram et al. (2014) considered the refractory period and inter-lymphangion contraction delay, they demonstrated that maximum pump flow in a multiple lymphangion model required different contraction timing variables for each lymphangion and complex valve actuation properties. This differs from Venugopal et al. (2007), who considered multiple lymphangions but did not recognize the importance of valve actuation.

Further studies by Bertram and colleagues analyzed extended networks of lymphatic collecting vessels with multiple lymphangions (Bertram et al. 2016a; b; Jamalian et al. 2016). Jamalian et al. (2016) considered a network of vessels with four inlets converging on one outlet, with each vessel composed of four lymphangions. Their results showed that under a wide range of adverse trans-axial pressure gradients (the usual physiological situation), networks of lymphatics with 10 lymphangions per vessel had the highest pumping capability. This can be considered the first instance of analyzing a symmetric branched lymphatic network with many contracting lymphangions. This computational platform is highly advantageous over experimental methods for analyzing the coordination of lymphatic vessels in a network, but it is challenging to experimentally validate due to its highly idealized vessel formation.

The interstitial pressure of many tissue beds is sub-atmospheric (Aukland and Reed 1993), and therefore, the mechanism of fluid uptake into initial lymphatics is not well understood. Jamalian et al. (2017) showed that the suction effect from contractions of downstream collecting vessels contributes to this fluid uptake. Using ex vivo collecting lymphatic data to validate their computational model, they showed that the suction effect was due to a transient drop in pressure downstream of the secondary valve after a contraction, which transmits into the initial lymphatic vessel, causing fluid inflow from the interstitial space. This suction effect of collecting vessels had not previously been substantiated.

Bertram et al. (2019) presented a minimal phenomenological model explaining the reduction in phasic contraction force and frequency in collecting lymphatic vessels resulting from flow-induced wall shear stress. Their model was applied to a previously validated numerical model of a phasically contracting lymphangion. Parameters of the Bertram et al. (2019) model were quantitatively matched to observations in isolated segments of rat mesenteric lymphatics and thoracic duct.

Razavi et al. (2017) investigated the correlation between lymphangion chain length and maximum pressure generation along a rat tail. Notably, they utilized in vivo near-infrared imaging to validate the model with effective pumping pressure measurements. Additionally, they conducted ex vivo experiments on isolated collecting vessels, revealing that the outflow pressure relies on the number of lymphangions in the chain and the force generated in lymphatic muscle cells. Importantly, the contractile frequency does not influence the resulting outflow pressure. Overall, this model provides a valuable platform for studying factors influencing fluid transport in a chain of lymphangions.

Morris et al. (2021) developed a fully coupled multiscale model of lymphatic pumping, including sub-cellular, cellular, and tissue-level mechanisms based on rat mesenteric lymphatics. None of the prior models considered multiscale mechanisms. These three levels were coupled via the lymph velocity inserted from a larger scale to a smaller scale, utilizing the contractile forces when upscaling. The excitation mechanism included in the model triggers periodic contractions. Results showed that the spontaneous calcium oscillations during diastole are responsible for increased outflow values. Morris et al. (2021) showed that their multiscale model produced similar results when compared with experimental data (Davis et al. 2011; Zawieja 2009).

There are limited computational models that simulate lymphatic function within different organs and disease states in the literature, and only six models were identified in this search (Caulk et al. 2016; Ngo et al. 2019; Possenti et al. 2019; Ashworth et al. 2023; Grotberg and Romanò 2022; Lavrova and Postnikov 2021). Ngo et al. (2019) published a study on pulmonary fluid balance to explain cardiogenic pulmonary congestion in humans. They were the first to incorporate a cardio-pulmonary model, interstitial fluid exchange determined by the Starling equation, and lymphatic pumping to study the fluid balance in the lung interstitium. They used an equivalent electrical representation of the Starling equation to model the lymphatic uptake from the interstitial space. Using this model, they demonstrated that continuous airway pressure is beneficial for interstitial fluid clearance.

Caulk et al. (2016) developed a model to study lymphedema, a disease characterized by tissue swelling due to dysfunctional lymphatic drainage. Their main contribution was the introduction of a theoretical framework for acute and long-term adaptation of lymphatic collecting vessels to study variations in mechanical loading such as pressure. They primarily modeled lymphedema by considering the geometric remodeling of the tissue and the reduction in lymphatic contractile function.

#### Continuum models

One-dimensional (1D) lymphodynamic models can be used to represent wave transmission effects and are therefore suitable for analyzing lymphodynamics in a network model as they can produce spatial information. There are only a few published 1D continuum models of the lymphatic system (Contarino and Toro 2018; Savinkov et al. 2020; Tretyakova et al. 2018). The first, developed by Reddy et al. (1977), attempted to model the lymphatic collecting network throughout the whole body. They used a single computational cell to represent lymphangions in the network considering only primary collecting vessels and showed resulting waveforms in different branches of lymphangions. However, flow rates in the thoracic duct showed many spikes, which is not physiologically realistic. Reddy’s lymphatic network model was too simple compared to the known network complexity, which consists of thousands of vessels (Suami 2017; Suami and Scaglioni 2018). Furthermore, Reddy’s results were not validated against experimental data because there were none available at the time.

In 2008, MacDonald et al. (2008) refined the model proposed by Reddy et al. (1977) by modeling flow through lymphangions and considering the bending and damping mechanisms of the vessel wall. Their model consisted of only a few lymphangions, in contrast to the model developed by Reddy et al. (1977). Importantly, they demonstrated that Reddy’s model was probably affected by numerical artifacts in wave propagation, which arose from the sensitivity to time step selection.

Contarino and Toro (2018) published a model of lymphangions considering dynamic contractions using an electro-fluid-mechanical contraction (EFMC) model. This EFMC model couples the electrical activity of lymphangions (action potentials) with fluid-mechanical feedback (circumferential stretch of the lymphatic wall and wall shear stress) and lymphatic vessel wall contraction. Further studies have focused on the geometrical/network characteristics of the human superficial lymphatic network. For example, Savinkov et al. (2020) used an anatomical data-based graph model to quantify the steady-state fluid balance in the lymphatic network, assuming a pressure–velocity relationship given by Poiseuille’s equation. This can be considered the first extended human lymphatic network model since the original study by Reddy et al. (1977).

A few studies have presented 2D lymphangion models. Li et al. (2019, 2022) published models including an initial lymphatic vessel and multiple collecting lymphangions with valves embedded in porous tissue. The model of Li et al. (2019) was similar to the work of Contarino and Toro (2018), where the contraction and relaxation of collecting vessels were passively affected by fluid pressure, while intracellular Ca^2+^ fluxes drove active contractions. Li et al.’s model showed strong nonlinear dynamics in the vessels, which agreed with experimental findings (Bertram and Davis 2023). Furthermore, when there was elevated tissue fluid pressure or reduced lymphatic pressure at the system’s outlet, it led to increased shear stress and higher levels of NO, which inhibits contractions. Later, Li et al. (2022) used their computational platform to explain the effects of gravitational forces on lymphatic drainage. They estimated the changes in lymphatic drainage when the gravitational force assisted or opposed flow.

Another 2D model of a chain of lymphangions was published by Elich et al. (2021). Fluid flow was modeled using the Navier–Stokes equation, and the immersed boundary method was utilized to achieve two-way coupling of the fluid–structure interaction (FSI). They investigated the effects of chain length, contraction style, and adverse axial pressure difference on cycle-mean flow rates. They achieved a good agreement with estimated results for valve dynamics, flow rates, pressure values, and vorticities in the valve-sinus region when compared to a range of previously published computational and experimental findings (Wilson et al. (2018), Davis et al. (2011), and Davis et al. (2012)).

In et al. (2021) conducted in vitro experimental and numerical investigations employing microfluidic devices. Their device replicated the characteristics of secondary lymphatic vessels, with flexible bicuspid valves, which they referred to as the "microfluidic valvular chip." They developed a FSI model and successfully emulated the flow properties observed within the microfluidic valvular chip. Furthermore, they estimated the impact of valve compliance and fluid viscosity on the resulting flow output and compared the velocity profiles to those found in arteries, revealing intriguing parallels, particularly under pathological conditions.

Eighteen studies have developed 3D models of lymphatics (Table 1), which can describe more complex geometry of the lymphangions and lymphodynamics when compared to 0D and 1D models. Rahbar and Moore (2011) modeled a single 3D lymphangion, neglecting the valves and inertia effects. A boundary condition was incorporated to model vessel wall movements. Hence, this model cannot be considered as a complete FSI model. Wilson et al. (2013) developed a 3D lymphatic model using confocal imaging data, focused on nitic oxide (NO) transport in rat mesenteric lymphatic vessels. They solved Navier–Stokes and mass transfer equations to estimate/simulate NO transport in the vessel, showing that NO concentration adjacent to the valve leaflets primarily resulted from a flow-mediated process rather than a sheer activated response of lymphatic endothelial cells. Meanwhile Jayathungage Don et al. (2021) focused on understanding the local mechanics and efficacy of fluid uptake into the lymphatic network. They developed a comprehensive 3D computational platform considering initial lymphatics, pre-collectors, collecting vessels, and the interstitium to better understand the lymphatic system as a drug delivery route.

Adeli Koudehi et al. (2023) presented a reconstructed 3D lymphangion model obtained through segmentation from micro-CT scans of the collecting lymphatics in the hind limb of mice. This was the first study to use 3D imaging to generate accurate structural information of multiple lymphangions, which moved beyond previous models that used idealized geometry. Their approach involved employing a FSI model to investigate the interaction between the contracting three-part lymphangion model and the surrounding poroelastic interstitium. Their results aligned with other computational and experimental studies (Bertram et al. 2011; Davis et al. 2011), particularly concerning valve actuation and the effects of outlet pressure on overall flow dynamics. Their research also showed that an increase in Young's modulus of the interstitial space and vessel wall had adverse effects on lymphatic drainage.

#### Secondary valve models

Most of the models available for secondary lymphatic valves are lumped parameter (0D) models (Table 1). Lymphatic secondary valve resistance is highly nonlinear with respect to transvalvular pressure difference (Bertram 2020). Therefore, the opening and closing intervalvular pressure drops are different. This nonlinearity in the valve resistance between the opening and closing states (hysteresis), where there is a bias to remain open, was experimentally shown by Davis et al. (2011). This happens particularly when the vessel is partially distended, which reduces resistance to flow. In addition, significant nonlinearity was identified in the lymphangion wall’s stress–strain relationship, where a significant change in stiffness occurs over a pumping cycle (Bertram et al. 2016a, b; Ikhimwin et al. 2020). This variation in stiffness is greater than comparable arterioles and venules (Moore Jr. and Bertram, 2018). Therefore, there are different opening and closing characteristics with both transvascular and transmural pressures (Davis et al. 2011).

The simplest computational models of secondary valves (Macdonald et al. 2008; Quick et al. 2007; Reddy et al. 1977) were open- or closed-type, and the backflow prevention method was prescribed in computational solvers. Here, the valves are fully open when there is a positive axial pressure gradient and vice versa. This approach has led to many discontinuities in the solutions. Motivated by this issue, Bertram et al. (2014) used smooth sigmoidal functions, where the valve resistance depends on parameters defined by Davis et al. (2011). In contrast, Contarino and Toro (2018) proposed a unique lymphatic model by modifying an existing blood vessel model with a predefined open and close time. Valves were modeled as a mechanobiological response based on the action potentials generated in the lymphatic endothelium and lymphatic muscle cells, which simulated the physiological characteristics of the system.

Material characteristics of the valve leaflet contribute to the hysteresis, and most computational models have not yet been able to simulate its function. Wilson et al. (2013) attempted to model its function by using confocal images to study both transient and steady-state flow through lymphatic valves. They estimated the wall shear stress distribution and NO generation, which is shear stress dependent. Subsequently, Wilson et al. (2015) expanded upon this model to investigate how alterations in the size of the sinuses and leaflets impact the deflections experienced by the valve leaflets and, consequently, the valve’s resistance to forward flow. Using an uncoupled FSI model, Wilson et al. (2015) estimated the resistance due to the presence of sinuses with valve leaflets and showed that its value is lower than the resistance of a straight tube without valves. Wilson et al. (2018) later improved this model by developing a coupled FSI model, using it to estimate the valve resistance, and showed it was 125% higher when compared to the previous model.

Bertram (2020) proposed a 3D FSI FEM model of the lymphatic valve actuation to model the hysteresis observed experimentally. Their 3D model was developed using confocal images with the fluid flow and the structural components solved simultaneously. Their estimated valve resistance agreed with the reported values of Wilson et al. (2015) when the vessel distension was ignored. Later, Bertram and Davis (2023) expanded the 3D model to simulate valve closure stages, addressing issues where adjacent leaflets crossed over during closure in their prior model. They also incorporated different material properties at locations where valve leaflets connect to the vessel walls. Bertram and Davis (2023) also generated diameter versus transmural pressure data upstream and downstream of valve leaflets using ex vivo murine lymphatic vessels. Notably, their findings revealed strong nonlinear properties of lymphatic vessels. They found that varying material properties at the connection point of the valve leaflets, upstream and downstream, did not impact valve closure. However, they observed a slight but noteworthy displacement of the center-line trailing edge when the upstream valve wall was more flexible, responding to adverse pressure differences.

Ballard et al. (2018) took a different numerical approach to model the secondary valve function. They used the lattice Boltzmann method to model the lymph flow and the lattice spring to capture the valve function. Their investigation revealed that shorter valve leaflets exhibit reduced flow resistance. However, when valves become excessively short, they fail to completely block backflow, suggesting a requirement for an optimal valve length. Later studies by the same group (Wolf et al. 2021, 2023) focused on the pumping dynamics of a peristaltically contracting collecting vessel with compliant valves. Wolf et al. (2021) showed that an optimum valve elasticity maximizes the pumping flow rate. Then following this, Wolf et al. (2023) investigated the effects of valve spacing with different contraction wavelengths on pumping performance using the same computational platform. They demonstrated that the mismatch between inter-valve distance and the wavelength of the peristaltic vessel motion results in increased pumping performance. However, to date, none of these 3D secondary valve models have been able to fully characterize and explain valve hysteresis.

### Lymph node models

Lymph nodes are located throughout the body along the course of lymphatic vessels and have a highly complex internal anatomical structure that impacts lymph flow through the node. Whilst afferent and efferent lymph node flow can be measured experimentally, it is particularly challenging to measure the flow dynamics within the lymph nodes themselves. However, the micro-architecture and its effects on the lymph flow can be investigated through computational modeling, which is significantly aided by using imaging techniques to give an accurate anatomical structure (Cooper et al. 2016; Jafarnejad et al. 2015).

Five studies developed CFD models of lymph flow within lymph nodes, all published within the last 10 years. First, Jafarnejad et al. (2015) used confocal microscopy to reconstruct the geometry of a mouse popliteal lymph node and FEM techniques to predict the resultant flow field. Their simulations showed that 90% of lymph fluid flow takes a peripheral path. They were also able to estimate the hydraulic conductivity value range in the medulla based on reported flow rate data of Adair and Guyton (1985), which is usually arduous to measure using in vivo methods. Cooper et al. (2016) also developed an FEM model to investigate the effects of internal structural properties on lymph flow (Fig. 2), using selective plane illumination microscopy to define the structure of a dog popliteal lymph node. Their model simulated lymph fluid taking a direct path from afferent to efferent lymphatic vessels, which is different to Jafarnejad et al. (2015) who showed lymph fluid moving to the center of the lymph node. They showed that passage of flow through the node is much slower compared to the peripheral route, which agrees with the findings of Jafarnejad et al. (2015). They also estimated the whole lymph node permeability based on the experimental flow rate values of Adair and Guyton (1985).

In 2022, Giantesio et al. (2022) published a 2D flow model considering a spherical idealized lymph node geometry. Their objective was to explain fluid flow within the lymph node, particularly along the peripheral path near the outer wall (subcapsular sinus), as opposed to penetration into the central lymphoid tissue compartment. They used the Stokes equation in the subcapsular sinus compartment (free fluid region) and Darcy’s law to model the flow. First, they obtained a numerical solution using the stream function and then compared the results with a FEM model. Giantesio et al. (2022) demonstrated that the resultant flow is pulsatile inside the subcapsular sinus compartment and 90% of the flow takes the peripheral path, which agreed with the earlier findings of computational studies (Jafarnejad et al. (2015); Cooper et al. (2016)).

Tretiakova et al. (2021) used the outcomes of Setukha and Tretiakova (2022)s’ work to develop a mathematical model of lymph nodes using neural networks (NNs). This was the first use of NN, which enable faster flow simulation results than mathematical models. Their developed mathematical model consisted of partial differential equations, including Darcy’s law and Starling’s equation, which explain the interstitial fluid flow and fluid balance in the blood and lymphatic system. Tretiakova et al. (2021) first created an equivalent representation of the system of PDEs using the boundary integral equation approach. Then, the numerical solution was obtained. Lastly, the NN model was trained to emulate the filtering function of the boundary integration model’s outcomes. Importantly, Tretiakova et al. (2021) showed the possibility of developing the NN model and obtaining faster prediction of lymph node drainage patterns and pharmacokinetics of drugs and circulation of other particles such as immune cells and cytokines.

Setukha and Tretiakova (2022) published a model considering the lymph filtration and absorption in a piecewise homogeneous domain. The modeling of lymph flow was accomplished using Darcy's law. Additionally, the modeling of lymph absorption, which includes fluid filtration from blood capillaries, was achieved by utilizing a boundary integral representation for velocity and pressure. This representation accounts for the resultant outflow through the absorption process. One of their main contributions was the development of a numerical scheme to solve the complex mathematical model which included boundary equations. They demonstrated good agreement between total outflow characteristics produced by simulation and the experimental data of Adair and Guyton (1985).

### Non-fluid dynamic models

There are additional non-fluid dynamic modeling approaches in the literature, including graph, mechanobiological, statistical, and agent-based models. For example, Tretyakova et al. (2018) developed a graph model to analyze key features of the 3D structural organization of the lymphatic collecting vessel network and assess geometrical parameters such as variations in branch length and nodal points. Mechanobiological models developed to date include those by Behringer et al. (2017) and Kunert et al. (2015) who modeled the biochemical properties of lymphatic endothelial cells within collecting lymphatic vessels.

Behringer et al. (2017) discovered a new signaling mechanism in lymphatic endothelial cells independent of calcium-activated potassium (K_Ca_) channels, which are typically found in arteries and play a crucial role in endothelial cell signaling. They also explained that the depolarization effects in endothelial cells facilitate the rapid conduction of contraction waves in lymphatic muscle cells. Kunert et al. (2015) used mathematical simulations to demonstrate that two opposing mechanobiological oscillators are sufficient to regulate fluid transport in the lymphatic system. Their simulations showed spatiotemporal alternations of Ca^2+^ and NO levels, forming feedback loops. These feedback loops generate phasic contractions that effectively propel lymph flow.

Statistical atlas-based models have also been developed by Reynolds et al. (2007, 2010) and Blumgart et al. (﻿^2011^) to analyze lymphatic drainage of the skin and breast, respectively. Their models were based on aggregated lymphoscintigraphy (LS) imaging data, from thousands of melanoma and breast cancer patients. Each patient had their primary tumor location and ‘sentinel’ lymph nodes (SLNs) identified, where an SLN is defined as any lymph node that directly drains a primary tumor site. Both Reynolds et al. (2010) and Blumgart et al. (2011) carried out comprehensive statistical analyses to test historical-based assumptions and provided updated atlases of superficial lymphatic drainage.

Numerous lymph node atlases have been constructed over the years to define the spatial distribution of lymph nodes throughout the body. These have typically been developed using 3D medical imaging data, including CT, MRI, SPECT/CT and PET/CT, often for the purpose of radiotherapy and surgical treatment planning (Harisinghani and O’Shea 2013). For example, the distribution of SLNs in breast cancer and prostate cancer patients was defined using SPECT/CT in studies by Novikov et al. (2021) and Ganswindt et al. (2011), respectively. Atlases quantifying the distribution of lymph node recurrence after cancer treatment have also been developed (Beaton et al. 2020). Work by Lee et al. (2013) developed 3D human anatomical models of the lymph node locations across ages and sexes for radiation dosimetry, using reference data. Other atlases have been developed for general purposes, such as the anatomical atlas of lymph nodes throughout the entire body created by Qatarneh et al. (2006). Their atlas was constructed by locating approximately 1200 lymph nodes that were visible in the high-resolution Visible Human Anatomical (VHA) imaging data set.

Bogle and Dunbar (2012) and Moreau et al. (2016) developed agent-based models to computationally analyze actions and interactions of different autonomous agents in a system, in this case the processes occurring within lymph nodes during an immune response. Whilst their models did not incorporate any fluid flow dynamics, they did simulate the expansion and contraction of T cell populations, chemotaxis and trafficking within the lymph node paracortex during an immune response. More recently, microimaging studies of a murine lymph node by Kelch et al. (2015, 2019) were combined with these agent-based models, giving complex 3D detail of the microvascular network within lymph nodes. They combined novel imaging and computational techniques to map the highly detailed conduit anatomy, which was used to simulate motility of T cells in different zones within the lymph node (Kelch et al. 2019).

## Current limitations and gaps

**Fig. 3:**
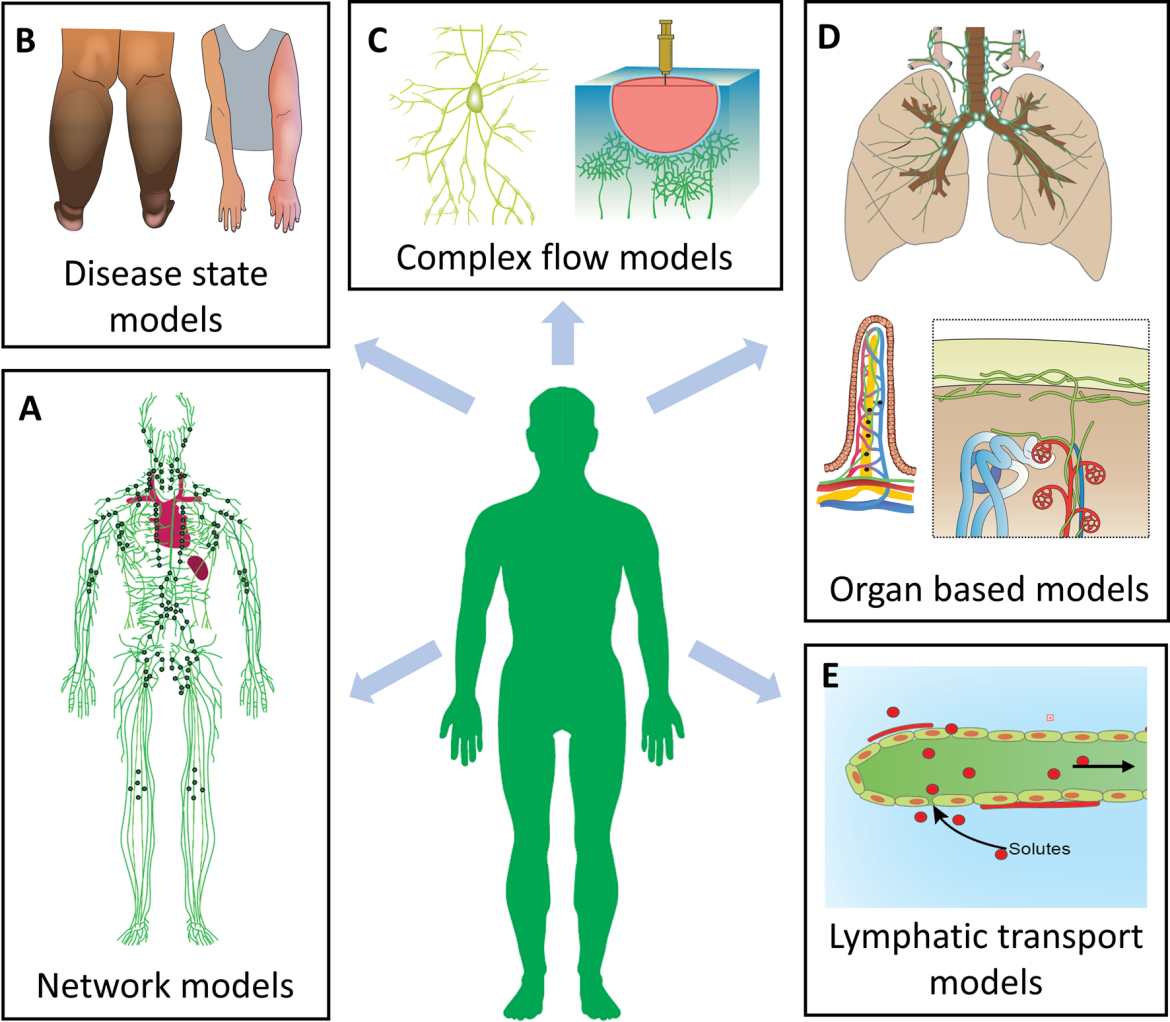
Computational models of the lymphatic system that are currently limited include models simulating: **A** flow through spatially distributed lymphatic networks; **B** disease states such as lymphedema and lymphatic filariasis; **C** complex lymph flow; **D** lymph flow in organs such as lacteals in the gut, and lymphatics in the lungs and kidney; and **E** transport of drugs, proteins, and cancer cells through the system. Part of image D modified with permission from Russell et al. (2019)

Various factors have contributed to the limited number of CFD models of the lymphatic system. Model validation remains particularly challenging and has been limited by the inherent challenges in obtaining structural and functional data. This is the main reason behind the numerical lack of “distributed models” compared to “lumped parameter models”, which can be developed with minimal data. Furthermore, most computational models have used ex vivo experimental data for validation; however, the normal physiological conditions and external forces (such as skeletal muscle contraction) are necessarily altered or absent during ex vivo experimental procedures. This can cause propagation of non-physiological data into model simulations (Scallan et al. 2016). Furthermore, most validation data come from animal rather than human studies, limiting its applicability for simulating the lymphatic system in humans. Figure 3 schematically shows key gaps identified within the field, which need to be addressed to advance the development of CFD models of the lymphatic system.

### Spatially distributed network models

Spatially distributed models explain variables of interest as a function of time as well as one or more spatial variables. In contrast, lumped parameter models explain variables of interest as a function of time only. There are currently anatomically correct spatially distributed network models of blood circulation (Blanco et al. 2020; Milanovic et al. 2021; Safaei et al. 2016), some of which consist of thousands of vessel segments (Blanco et al. 2020). However, similar models do not exist for the lymphatic system because most of the focus has been on lumped parameter models. As previously described, Reddy et al. (1977) developed a simplified model of the lymphatic network but only considered the larger vessels, which was later shown to give inaccurate results as it was oversimplified (Macdonald et al. 2008). An anatomically accurate and detailed network model would provide an important framework for studying dermal or deeper level lymphodynamics throughout the whole body.

### Disease state models

Disease state models of the lymphatic system are also rare, with only five in Table 1 focusing on lymphedema and lung lymphatics (Ashworth et al. 2023; Caulk et al. 2016; Ngo et al. 2019; Possenti et al. 2019; Grotberg and Romanò 2022). Recent advances in knowledge regarding lymphatic physiology have revealed its importance in a variety of disease states, including cardiovascular disease, multi-organ failure, cancer metastasis, pulmonary edema and obesity (Itkin 2018; Itkin et al. 2021; Russell et al. 2019; Singhal et al. 2023). Lymphatic dysfunction and its adverse effects can be studied and analyzed more efficiently in a computational platform compared to an experimental model. Furthermore, it is not always possible to directly measure lymphatic function in vivo deep within organs such as the liver or kidney. Modeling pathological scenarios in organ-specific lymphatic models would be a valuable tool for understanding conditions such as edema, systemic inflammatory response syndrome (SIRS), and multi-organ dysfunction syndrome (MODS) (Itkin et al. 2021).

### Complex flow models

Most lymphatic models assume laminar flow through the lumen of collecting vessels. However, lymphatic imaging studies have shown that there are frequent changes in the length-to-diameter ratio throughout collecting vessels and hence, that a laminar flow assumption may not be valid (Margaris and Black 2012). In addition, lumped parameter models do not include spatial information, which is problematic when attempting to explain localized effects. For example, secondary valve closure can create unsteady flow properties near the valve, which can be studied using a more detailed spatial description of the flow. Lymphatic pumping and lymph formation are also influenced by passive forces such as arterial pulsation, skeletal muscle movement and ECM changes, which are predominantly localized. Thus, more complex flow descriptions are required to capture this level of complexity.

### Organ-specific models

Despite many models using parameters from organ systems (such as the mesentery), organ-specific models of the lymphatic system are limited. For example, there are only three published studies of the pulmonary lymphatic system (Ashworth et al. 2023; Ngo et al. 2019; Grotberg and Romanò 2022). In recent years, there has been a notable surge in interest surrounding the meningeal system. Nevertheless, this remains a developing field within neuroscience, and a comprehensive understanding of its physiology is still a working progress (Da Mesquita et al. 2018; Louveau et al. 2015). Further research is therefore required to elucidate the contribution of lymphatics to organ function, especially for critical organs such as the lung, heart, gut, and kidney (Bernier-Latmani and Petrova 2017; Russell et al. 2019, 2023). This process has been hampered by the limited anatomical and physiological data required to build lymphatic models for these organ-based systems, which presents significant challenges, but also opportunities for rapid progress in the field.

### Transport models

Lymphatic transport models are limited in the literature and would be beneficial for understanding the transport of drugs, proteins, or cancer cells through the lymphatic system. Novel drugs, such as lipid particles and lipid-based nanostructured drug carriers, can be delivered via the lymphatics to provide added advantages, including direct targeting of disease progression (e.g., cancer, HIV) and bypassing the first-pass metabolism in the liver (Jayathungage Don et al. 2021; Trevaskis et al. 2015). As discussed earlier, drug transport has received limited attention in existing computational modeling studies. Many factors, such as drug particle shape, drug size, interstitial properties, and hydrophobicity, regulate lymphatic drug delivery. Since there are many novel drugs and the lymphatic system plays a role in various diseases, drug transport via the lymphatic system can be studied in a computational platform. This platform could be used to enhance and optimize drug delivery systems. Experimental particle transport data from imaging studies, such as lymphoscintigraphy, could be leveraged to develop such models.

## Conclusions

Over the past decade, lymphatic research and the development of in silico models of lymph flow dynamics have gained momentum. However, substantial work is required to match its cardiovascular system counterpart. This review summarized existing lymphatic computational models, with a particular emphasis on lymph fluid transport models. Additionally, it has identified current gaps in the field that would benefit from focused attention moving forward. Given the intricate nature of the lymphatic system's active and passive mechanical responses, realistic computational models require a multi-physics and multidisciplinary approach. There are optimistic signs that further efforts over the next decade to characterize lymphatic anatomy and function, via experimental and imaging-based approaches, will provide key data to accurately inform and validate lymphatic CFD models. Such models will have the potential to enhance the understanding of the lymphatic system in both health and disease.
